# Usage and attitude of medical students towards mobile medical applications during and after COVID-19 lockdown: repeated cross-sectional study

**DOI:** 10.1186/s12909-024-05216-5

**Published:** 2024-03-04

**Authors:** Marwa Ahmed Alsharif, Ammar Ayman Bahbah, Mohamed Mabrouk Ghonaim, Janna Ahmed Omran, Maram Mohamed Rihan, Mariam Ahmed Zayed, Mariam Salah Tabour, Mariam Hamed Alwardany, Omar Ali Aboshady

**Affiliations:** 1 Faculty of Medicine, Menoufia University, Menoufia, Egypt; 2 Clinical Pharmacology Department, Faculty of Medicine, Menoufia University, Menoufia, Egypt

**Keywords:** Medical applications, Medical students, Medical education, COVID-19’ lockdown, Mobile apps

## Abstract

**Background:**

COVID-19 pandemic has accelerated the shift toward e-learning, particularly in medical education. Mobile medical applications (apps) have become integral tools for e-learning due to the prevalence of smartphones among medical students. Therefore, we aim to assess the usage and attitude of undergraduate Egyptian medical students towards mobile medical apps during and after the COVID-19 lockdown.

**Methods:**

This is a two-phase repeated cross-sectional study using an online, pilot-tested, and self-administered survey conducted at the Faculty of Medicine, Menoufia University, Egypt. Phase 1 was during the academic year 2019–2020 (during lockdown), and phase 2 was during the academic year 2021–2022 (after lockdown). Out of the 4800-target population for each phase, a sample size of 140 medical students was randomly selected from each study year, resulting in a total sample size of 840 students for all six academic and clinical years for each phase.

**Results:**

A total of 566 students in phase 1 and 616 students in phase 2 responded to the survey, with response rates of 67.62% and 73.33%, respectively. In phase 1, 55.7% of students reported using medical apps, with no significant difference between males and females (60.1% vs. 53.8%; *p* = 0.17) or between academic and clinical years (56.3% vs. 54.7%; *p* = 0.7). In phase 2, the percentage increased to 70.9%, with a significant difference between males and females (80% vs. 62.9%; *p* < 0.001) and between academic and clinical years (79.8% vs. 63%; *p* < 0.001). Medical dictionaries were the most commonly used apps, whereas medical calculators were the least common in both phases. Regarding their attitude, most students (65.1% and 73.9% in phases 1 and 2, respectively) expressed confidence in using medical apps, while 81.3% in phase 1 and 88.1% in phase 2 agreed that using medical apps is a flexible method of learning. Lack of knowledge regarding which app to download was the most reported cause of limitation in medical app usage by 37.8% of the students in phase 1 and 40% in phase 2.

**Conclusion:**

Our study revealed that the COVID-19 lockdown led to a significant increase in the use of mobile medical apps among Egyptian medical students. Despite the positive attitude of students towards these apps, multiple challenges still need to be addressed to ensure their optimal utilization in medical education.

## Introduction

COVID-19 pandemic and subsequent lockdowns have profoundly impacted medical education [1]. In-person classes and clinical rotations were severely affected, leading schools to quickly adopt remote teaching methods such as online lectures and virtual patient simulations [2]. Since then, the medical education framework has undergone a substantial shift to accommodate new social distancing policies and to introduce a hybrid learning system [3]. However, implementing this system necessitated the use of novel technologies, which posed challenges for some students.

Medical applications “apps”, as versatile and effective tools for communication and medical education, have been suggested to play a crucial role in facilitating these adaptations [4, 5]. The development of mobile medical apps has further expanded the potential of smartphones and enhanced various aspects of healthcare, including learning, diagnosis, and decision making [6]. These apps are emerging as valuable supplements to traditional learning environments, providing medical students with convenient access to up-to-date medical information and expertise [7]. A recent systematic review and meta-analysis reported that mobile apps serve as valuable tools for enhancing knowledge and skill levels among healthcare professionals and students alike [8].

In developed countries, a UK survey revealed that 79% of medical students owned smartphones, with 78.3% using medical apps for educational purposes [9]. Similarly, a Canadian study reported that 85% of medical students utilized their mobile devices for medical learning at least once daily [10]. The Middle Eastern countries also showed comparable usage rates. For example, a 2016 study in Saudi Arabia found that approximately 89.1% of students used medical apps, although most were unaware of the significance of apps in the learning process [11].

While studies have documented the use of various apps, such as surgical simulation apps, online meeting apps such as Zoom and Microsoft Teams, and virtual surgery educational programs, no studies have evaluated the status, attitudes, and limitations of these apps during and after the COVID-19 lockdown, particularly in developing countries [4, 5]. Therefore, the main objective of this study is to assess the percentage of medical app usage among undergraduate medical students in Egypt during and after the COVID-19 lockdown. In addition, we aim to assess students’ attitudes toward and perceptions of medical apps.

## Methods

A pilot-tested, self-administered, survey-based repeated cross-sectional study was conducted to assess the prevalence and attitude of medical students towards the use of medical apps during and after the COVID-19 lockdown at the Faculty of Medicine, Menoufia University, Egypt. Ethical approval was obtained from the Institutional Review Board of the Faculty of Medicine, Menoufia University, Egypt.

### Population and sampling

The target population for this study was undergraduate medical students in academic and clinical years during the academic years 2019–2020 and 2021–2022, excluding non-Egyptians and students in their internship years. The approximate total number of students included in the target population was approximately 4800 across all years. To achieve a 99% confidence interval, 5% margin of error, and 50% response distribution, a sample of 581 participants was required for each study time point, with an additional 45% added as a non-response rate, resulting in a total sample size of 840 students for each phase. The sample was evenly divided equally between all study years, with 140 participants randomly selected from each academic and clinical class based on the lists of registered student names.

### Data collection

For each year of study, at least one data collector was recruited to form teams and obtain lists of students for each class from official sources. The randomly selected sample from the students’ lists was invited via email or social media by receiving a detailed message with the required information about the study, a specific code for each participant, and a link to the questionnaire. After achieving the desired response rate, data were collected on a spreadsheet and accessed only by the investigators. The same data collection steps were applied to both phases. Phase 1 was during the lockdown from September to November 2020 (Egypt recorded its first confirmed case of COVID-19 on February 14, 2020 [12], and the government suspended face-to-face education across all medical schools on March 14, 2020, and introduced online education as a substitute [13]), and phase 2 was from July to August 2022 after the end of the lockdown.

### Questionnaire development

The study questionnaire was developed in English by the principal investigators and reviewed by two experts in questionnaire development. A pilot study was conducted with 40 participants, representing all academic and clinical years, to obtain feedback on the format, clarity, and completion time of the survey. Based on this feedback, revisions were made to improve the clarity of certain questions. The responses from the pilot study were not included in the final analysis.

The questionnaire was administered via Google Forms in both phases, with three additional questions added in phase 2 to assess the long-term effect of COVID-19 on app usage. The questionnaire consisted of 35 questions divided into six sections: (1) a cover letter providing information on the study aims, completion time, and consent to participate; (2) six questions on socio-demographics; (3) two questions about the type of mobile phone and Internet access; (4) 15 questions on the prevalence and use of medical apps before and after COVID-19; (5) 11 questions on attitudes towards the use of medical apps in medical education; and (6) one question on the limitations of medical app usage.

Most of the questions were multiple-choice with a single correct answer, whereas two questions allowed multiple responses via checkboxes. A five-point Likert scale was used to measure participants’ attitudes toward app usage in medical education, ranging from “strongly agree” to “strongly disagree,” and another scale was used to assess Internet speed, ranging from “very slow” to “very rapid.”

### Statistical analysis

Descriptive statistics are presented as frequencies and percentages. We used chi-square test to assess the association between qualitative variables. To present clear opinions in a simplified manner, the five-point Likert scale was consolidated into three categories: agree, neutral, and disagree. Similarly, the Internet speed scale was consolidated into three categories: slow, average, and rapid. The class year was recorded as a dichotomous variable to enable comparison between academic and clinical students. All tests conducted were bilateral in nature, and a p-value of 0.05 was used as the margin for statistical significance. Statistical analysis was performed using IBM SPSS Statistics for Windows version 28 (IBM Corp., Armonk, N.Y., USA), and the data were visualized using GraphPad Prism 9 statistical software (GraphPad Software, Inc., San Diego, CA, USA).

## Results

### Characteristics of participants

Of the 840 individuals contacted for each phase, 566 (67.62%) and 616 (73.33%) valid responses were received for phases 1 and 2, respectively. The gender distribution varied between the two phases, with females constituting the majority of phase 1 participants (71.3%), particularly in academic years (57.1%). In contrast, females made up 52.9% of phase 2 participants, with fewer participants from academic years (47.4%). Across both phases, 82.15% of the respondents reported using Google Android smartphones, while only 14.21% reported using Apple iOS (iPhones) (Table 1).

**Table 1:** Demographic characteristics of the respondents

	Phase 1 (*n* = 566)	Phase 2 (*n* = 616)
**Gender, n (%)**		
Male	163 (28.8%)	290 (47.1%)
Female	403 (71.2%)	326 (52.9%)
**Year of study, n (%)**		
Academic	323 (57.1%)	292 (47.4%)
Clinical	243 (42.9%)	324 (52.6%)
**Type of smartphone, n (%)**		
Google Android	475 (83.9%)	496 (80.5%)
Apple iOS (iphone)	70 (12.4%)	98 (15.9%)
Others	21 (3.7%)	22 (3.6%)
**Internet speed, n (%)**		
Watching videos		
Slow	109 (19.3%)	77 (12.5%)
Average	329 (58.1%)	368 (59.7%)
Rapid	128 (22.6%)	171 (27.8)
Social media		
Slow	39 (6.9%)	38 (6.2%)
Average	287 (50.7%)	321 (52.1%)
Rapid	240 (42.4%)	257 (41.7%)
Surfing websites		
Slow	110 (19.4%)	107 (17.4%)
Average	309 (54.6%)	334 (54.2%)
Rapid	147 (26%)	175 (28.4%)
Uploading		
Slow	248 (43.8%)	267 (43.3%)
Average	267 (47.2%)	272 (44.2%)
Rapid	51 (9%)	77 (12.5%)

**Fig. 1:**
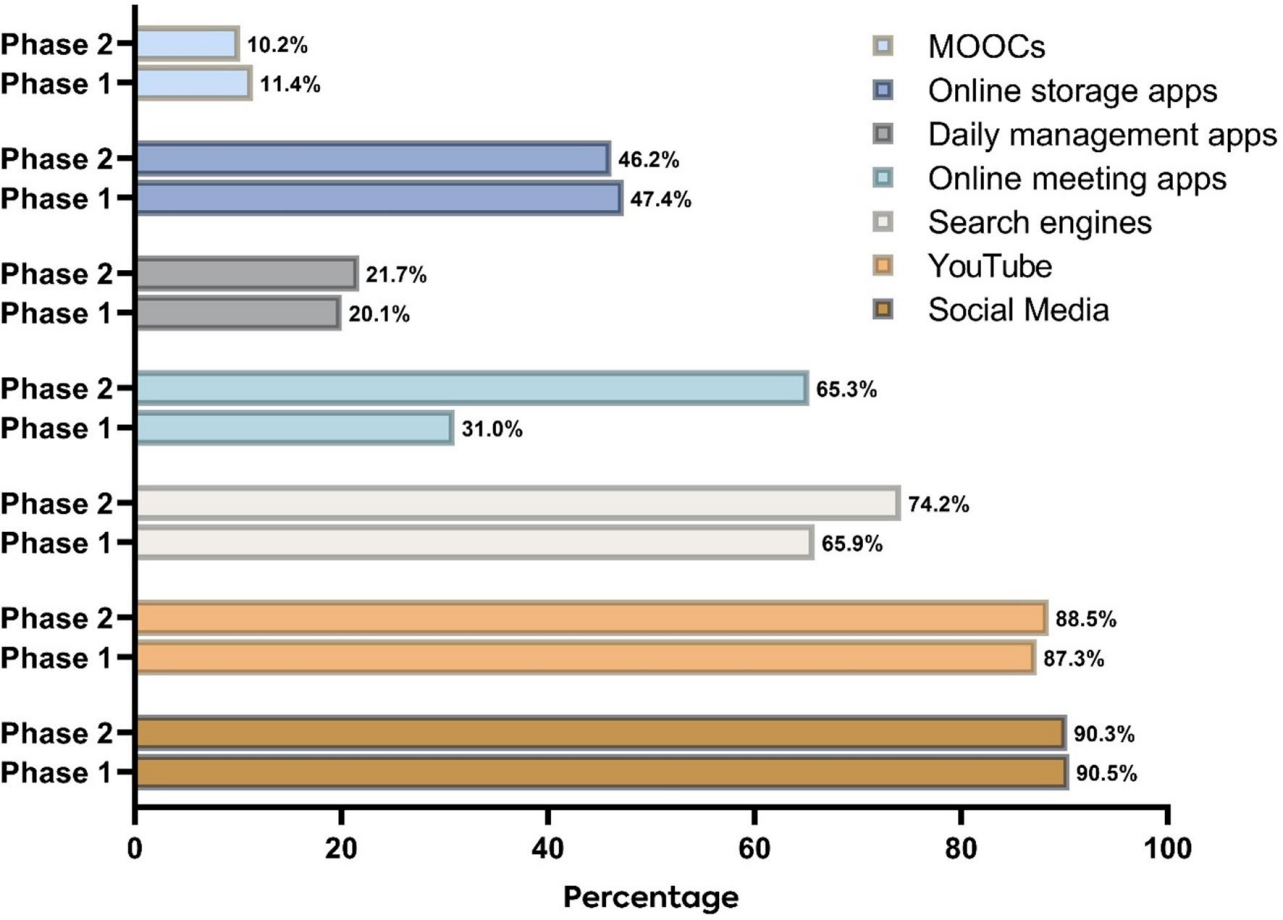
Mobile apps’ use during phase 1 and phase 2

The majority of the participants rated their Internet speed as an average for watching videos, social media, and browsing websites across both phases. However, more participants reported slow uploading Internet speeds in both phases (Table 1). The use of social media apps and YouTube remained consistently high across both phases, with approximately 90% of the participants reporting usage. Notably, the usage of online meeting apps significantly increased in phase 2, with 65% of participants reporting usage compared to only 31% in phase 1 (Fig. 1).

### Usage of medical apps

In phase 1, 55.7% of students reported using medical apps, with no significant difference observed between males and females (60.1% vs. 53.8%; *p* = 0.17) or between academic and clinical years (56.3% vs. 54.7%; *p* = 0.7). The number of app users increased to 70.9% in phase 2, with a significant difference noted between males and females (80% vs. 62.9%, *p* < 0.001) and between academic and clinical years (79.8% vs. 63%, *p* < 0.001). In phase 1, only 17.5% of students started using medical apps after the onset of COVID-19, with no significant difference between academic and clinical years (12.2% vs. 19.8%, *p* = 0.1) or between males and females (15.4% vs. 20.3%, *p* = 0.26). Interestingly, in phase 2, 45.5% of the students began using medical apps after the COVID-19 pandemic. While no significant difference was observed between males and females (43.5% vs. 47.8%, *p* = 0.37), a significant difference was found between the academic and clinical years (68.2% vs. 19.6%, *p* < 0.001). Moreover, of those who reported using apps before COVID-19 in phase 2, 64.3% reported an increase in app usage after the COVID-19 lockdown.

With regard to the number of medical apps used, the majority of students (88.3%) used five or fewer apps in phases 1 and 2. During phase 1, medical dictionary apps were the most commonly used apps among students in both academic and clinical years, with 86.8% and 76.6% reporting usage, respectively. Conversely, medical calculator use was the least popular among academic and clinical students, with 81.9% and 78.9% reporting never having used it before, respectively. This trend persisted in phase 2, with medical dictionaries remaining the most commonly used app and medical calculator usage remaining the least popular among students (Table 2).

**Table 2:** Types of used medical apps among academic and clinical years during phase 1 and phase 2

		Phase 1				Phase 2			
		Total (*n* = 315)	Academic (*n* = 182)	Clinical (*n* = 133)	P value	Total (*n* = 437)	Academic (*n* = 233)	Clinical (*n* = 204)	P value
Disease management apps	Never used before	95 (30.1%)	56 (30.8%)	39 (29.3%)	0.49	217 (49.6%)	155 (66.5%)	62 (30.4%)	**0.0001**
	Yearly	72 (22.8%)	47 (25.8%)	25 (18.8%)		94 (21.5%)	34 (14.6%)	60 (29.4%)	
	Monthly	98 (31.1%)	54 (29.7%)	44 (33.1%)		82 (18.8%)	25 (10.7%)	57 (27.9%)	
	Weekly	43 (13.6%)	22 (12.1%)	21 (15.8%)		34 (7.8%)	12 (5.2%)	22 (10.8%)	
	Daily	7 (2.2%)	3 (1.6%)	4 (3.0%)		10 (2.3%)	7 (3.0%)	3 (1.5%)	
Procedure guide apps	Never used before	171 (54.3%)	134 (73.6%)	37 (27.8%)	**0.0001**	161 (36.8%)	131 (56.2%)	30 (14.7%)	**0.0001**
	Yearly	75 (23.8%)	29 (15.9%)	46 (34.6%)		134 (30.7%)	49 (21.0%)	85 (41.7%)	
	Monthly	44 (14%)	10 (5.5%)	34 (25.6%)		108 (24.7%)	36 (15.5%)	72 (35.3%)	
	Weekly	19 (6.03%)	8 (4.4%)	11 (8.3%)		30 (6.9%)	14 (6.0%)	16 (7.8%)	
	Daily	6 (1.9%)	1 (0.5%)	5 (3.8%)		4 (0.9%)	3 (1.3%)	1 (0,5%)	
Medical Dictionaries	Never used before	55 (17.5%)	24 (13.2%)	31 (23.3%)	**0.01**	69 (15.8%)	41 (17.6%)	28 (13.7%)	0.055
	Yearly	71 (22.5%)	34 (18.7%)	37 (27.8%)		96 (22%)	40 (17.2%)	56 (27.5%)	
	Monthly	74 (23.5%)	45 (24.7%)	29 (21.8%)		118 (27%)	60 (25.8%)	58 (28.4%)	
	Weekly	79 (25%)	54 (29.7%)	25 (18.8%)		99 (22.6%)	58 (24.9%)	41 (20.1%)	
	Daily	36 (11.4%)	25 (13.7%)	11 (8.3%)		55 (12.6%)	34 (14.6%)	21 (10.3%)	
Lab References	Never used before	167 (53%)	88 (48.4%)	79 (59.4%)	**0.027**	224 (51.2%)	122 (52.4%)	102 (50.0%)	0.051
	Yearly	85 (27%)	54 (29.7%)	31 (23.3%)		106 (24.2%)	45 (19.3%)	61 (29.9%)	
	Monthly	41 (13%)	31 (17.0%)	10 (7.5%)		65 (14.9%)	39 (16.7%)	26 (12.7%)	
	Weekly	18 (5.7%)	7 (3.8%)	11 (8.3%)		32 (7.3%)	22 (9.4%)	10 (4.9%)	
	Daily	4 (1.3%)	2 (1.1%)	2 (1.5%)		10 (2.3%)	5 (2.1%)	5 (2.5%)	
Drug Index	Never used before	131 (41.6%)	89 (48.9%)	42 (31.6%)	**0.0001**	182 (41.6%)	113 (48.5%)	69 (33.8%)	**0.008**
	Yearly	53 (16.8%)	31 (17.0%)	22 (16.5%)		81 (18.5%)	39 (16.7%)	42 (20.6%)	
	Monthly	71 (22.5%)	44 (24.2%)	27 (20.3%)		83 (19%)	35 (15.0%)	48 (23.5%)	
	Weekly	41 (13%)	13 (7.1%)	28 (21.1%)		66 (15.1%)	37 (15.9%)	29 (14.2%)	
	Daily	19 (6%)	5 (2.7%)	14 (10.5%)		25 (5.7%)	9 (3.9%)	16 (7.8%)	
Medical Calculators	Never used before	254 (80.6%)	149 (81.9%)	105 (78.9%)	**0.015**	338 (77.3%)	182 (78.1%)	156 (76.5%)	0.36
	Yearly	35 (11.1%)	25 (13.7%)	10 (7.5%)		52 (11.9%)	23 (9.9%)	29 (14.2%)	
	Monthly	18 (5.7%)	7 (3.8%)	11 (8.3%)		27 (6.2%)	15 (6.4%)	12 (5.9%)	
	Weekly	6 (1.9%)	1 (0.5%)	5 (3.8%)		17 (3.9%)	12 (5.2%)	5 (2.5%)	
	Daily	2 (0.6%)	0 (0.00%)	2 (1.5%)		3 (0.7%)	1 (0.4%)	2 (1.0%)	

* Bold *p* value means statistically significant

### Payment for medical apps

In phase 1, only a small proportion of students (3.5%) reported having paid for a medical app subscription. Notably, this percentage increased to 10.1% in phase 2. In phase 1, students who had subscribed to medical apps reported that these apps enabled active participation in class discussions (*p* = 0.001) and access to a large volume of information beyond that provided by their school library (*p* = 0.01). Similarly, in phase 2, these students indicated that medical apps provided an advantage in their medical studies (*p* = 0.008).

Interestingly, students who had never paid for a subscription reported that their devices could not support installing new apps (*p* < 0.001). In both phases of the study, the majority of students expressed a willingness to pay for an app subscription if they found it useful (83.2% in phase 1 and 82.6% in phase 2). Phase 1 students believed that using medical apps would help them complete their assignments (*p* = 0.012), while phase 2 students thought that the apps would increase their participation in class discussions (*p* = 0.004).

### Attitude toward medical apps

In phases 1 and 2, 65.1% and 73.9% of students, respectively, expressed confidence in using medical apps during their studies (Table 3). In phase 1, students who had started using medical apps before the COVID-19 pandemic reported higher confidence levels than those who had not (68.8% vs. 47.3%, *p* = 0.005). However, this difference was not significant in phase 2 (75.6% vs. 71.9%, *p* = 0.25). No significant difference was observed between academic- and clinical-year students in their confidence in medical app usage in either phase (phase 1:67.7% in academic years vs. 65.1% in clinical years, *p* = 0.33; phase 2:73% in academic years vs. 75% in clinical years, *p* = 0.56).

**Table 3:** Attitudes of medical students towards medical apps during phase 1 and phase 2

	Disagree, n (%)			Neutral n (%)			Agree n (%)	
	Phase 1	Phase 2	Phase 1		Phase 2	Phase 1		Phase 2
I feel confident using Medical Apps” in my personal medical studies	17 (5.4%)	20 (4.6%)	93 (29.5%)		94 (21.5%)	205 (65.1%)		323 (73.9%)
Using “Medical Apps” can increase my participation in class discussion	19 (6%)	33 (7.6%)	64 (20.3%)		99 (22.7%)	232 (73.7%)		305 (69.8%)
I feel the advantage of using “Medical Apps” in my medical study	7 (2.2%)	17 (3.9%)	55 (17.5%)		72 (16.5%)	253 (80.3%)		348 (79.6%)
“Medical Apps” help me to complete my assignments (tutorials, research)	17 (5.4%)	22 (5%)	65 (20.6%)		84 (19.2%)	233 (74%)		331 (75.7%)
Use of “Medical Apps” makes me independent in my studies	106 (33.7%)	101 (23.1%)	83 (26.3%)		123 (28.1%)	126 (40%)		213 (48.7%)
I think “Medical Apps” are not useful for learning	280 (88.9%)	380 (87%)	21 (6.7%)		24 (5.5%)	14 (4.4%)		33 (7.5%)
I can access a large volume of information on “Medical Apps” quickly via the Internet more than my school library	111 (35.2%)	27 (6.2%)	78 (24.8%)		64 (14.6%)	126 (40%)		346 (79.2%)
“Medical Apps” usage in class causes a social gap between lecturers and learners	47 (14.9%)	118 (27%)	46 (14.6%)		128 (29.3%)	222 (70.5%)		191 (43.7%)
Learning through “Medical Apps” is a flexible method of learning as it can be done anywhere & anytime	10 (3.2%)	7 (1.6%)	49 (15.6%)		45 (10.3%)	256 (81.3%)		385 (88.1%)
I think using medical apps has been increased after COVID-19 among medical students	-	20 (4.6%)	-		50 (11.4%)	-		367 (84%)
Using medical apps has increased the awareness toward COVID-19 pandemic	-	19 (4.3%)	-		111 (25.4%)	-		307 (70.3%)

There was no significant association observed between the number of medical apps used and students’ confidence in using medical apps in both phase 1 (*p* = 0.10) and phase 2 (*p* = 0.38). The majority of students in both phases reported the advantages of using medical apps in their studies, including flexibility (81.3% in phase 1 and 88.1% in phase 2) and assistance in completing assignments (74% in phase 1 and 75.5% in phase 2). However, only 40% of students in phase 1 believed that medical apps made them independent in their studies, while this percentage increased to 48.7% in phase 2.

The perceptions of medical students regarding medical apps have evolved over time, with only 40% perceiving them as providing quick access to a large volume of information in phase (1) This percentage increased significantly to 79.2% in phase (2) Additionally, the perception of medical apps causing a social gap between lecturers and learners decreased markedly, from 70.5% in phase 1 to 43.7% in phase 2. In phase 1, belief in the social gap was significantly associated with academic grade (*p* < 0.001) and the number of medical apps used (*p* = 0.027). However, these associations were not significant in phase 2. Interestingly, in phase 2, 84% of the participants believed that medical app usage had increased among medical students after the COVID-19 outbreak, and 70.3% thought that medical apps had raised awareness about the pandemic.

### Limitations for medical app usage

**Fig. 2:**
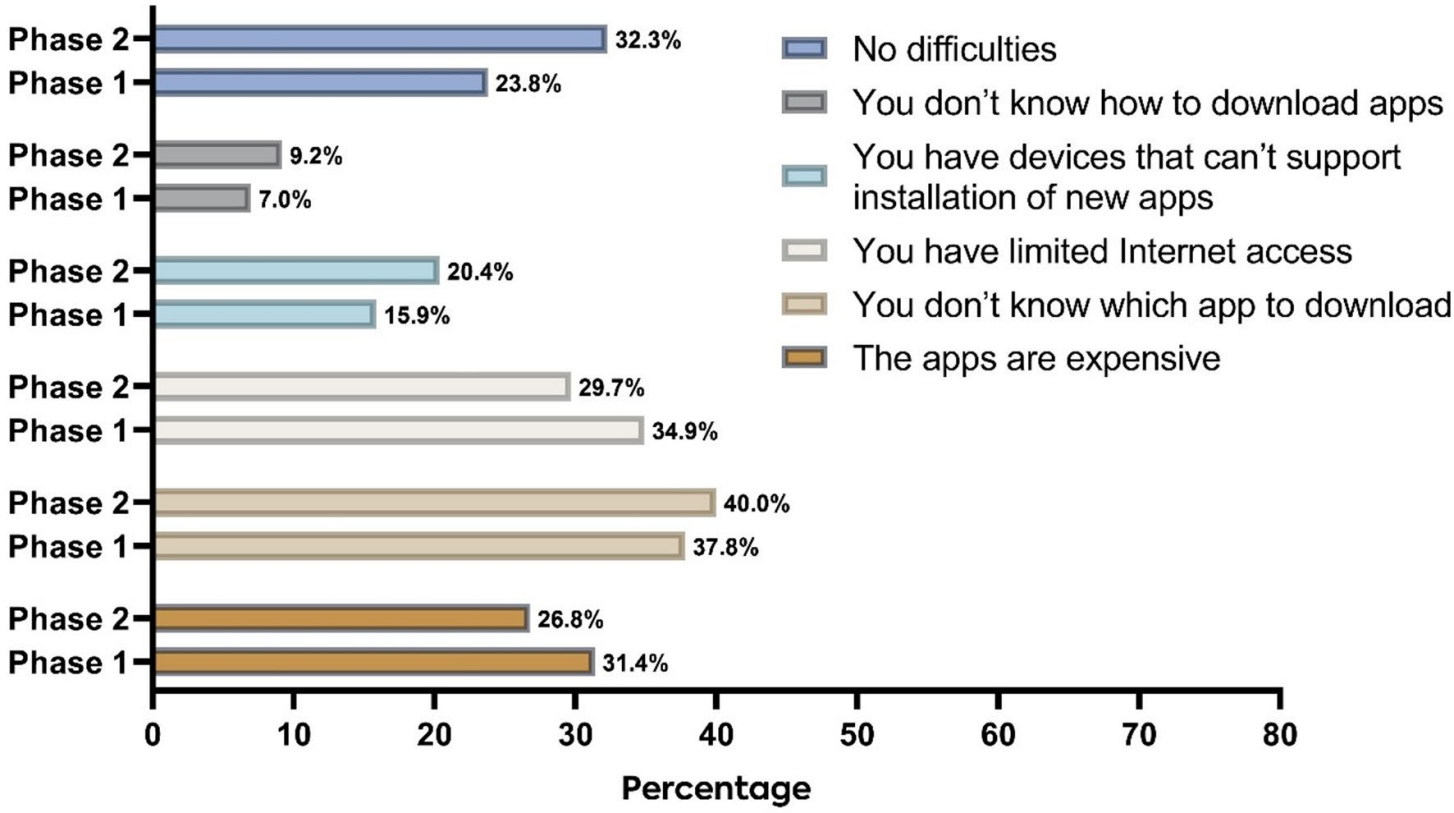
Limitations of using medical apps during phase 1 and phase 2

Among the students surveyed, 23.8% and 32.3% reported facing no difficulty using medical apps in phase 1 and phase 2, respectively. However, in phase 1, the most common limitation reported was a lack of knowledge regarding which app to download, with 37.8% of students citing this as a challenge. This limitation was more prevalent among the female students (43.3% vs. 25.5%, *p* = 0.003). Similarly, in phase 2, the most common limitation reported was still a lack of knowledge regarding which app to download, with 40% of students citing this as a challenge. This limitation was also more common among the female students (48.8% vs. 32.3%, *p* < 0.001). Additionally, students in academic years were more susceptible to this limitation than those in clinical years (45.1% vs. 34.3%, *p* = 0.02) and faced more limited Internet access (36.5% vs. 22.1%, *p* < 0.001). The remaining limitations of medical apps are shown in Fig. 2.

## Discussion

Our study found a significant increase in the use of medical apps following the COVID-19 pandemic, with overall positive attitudes and perceptions towards using these apps in medical education. This trend is reflected in the rise in apps’ usage from 55.7% in phase 1 to 70.9% in phase 2. One possible explanation for this increase is the mandatory implementation of distance e-learning in Egyptian schools and universities during the lockdown, which may have motivated students to explore new sources of information and compensate for missed university attendance [13]. Additionally, the availability of spare time may have contributed to the amplified usage of medical apps.

It is noteworthy that in phase 1, no significant difference was observed between male and female medical students in terms of medical app usage. However, in the second phase, a significant difference in favor of males was observed, at a rate of 80%. When compared to other studies, a study conducted in the UK in 2012 on 257 medical students found that 79.9% of smartphone users owned medical apps, and similar to our results, males were more likely to possess medical apps [9]. Another study conducted by Sayedalamin et al. at King Abdulaziz University in Saudi Arabia reported that 99.4% of medical students owned smartphones and 89.1% had installed medical apps, with no significant difference based on gender [11].

No significant difference was found between academic- and clinical-year students in phase (1). However, in phase 2, academic-year students utilized medical apps more than clinical students. This finding is inconsistent with another study conducted in Pakistan on 448 medical students, which showed that fifth-year students used the apps more than second-year students (93% and 62%, respectively) [7]. In relation to the number of medical apps, the majority of students reported owning between one and five apps in phase 1, and this proportion remained consistent in phase (2). This aligns with a previous study conducted in the UK, which found that nearly half of the medical students used between one and five medical apps [14].

### Attitude toward medical apps

A positive attitude towards confidence in the use of medical apps was observed among 65% and 73.9% of the respondents in phases 1 and 2, respectively, irrespective of the number of apps used. Furthermore, a comparable percentage of respondents in both phases acknowledged the flexibility offered by medical apps as a means of learning. Interestingly, only 40% of phase 1 respondents agreed that the use of medical apps enables them to access a large volume of information compared to the traditional school library. In phase 2, the percentage increased significantly to 79.2% after the application of the hybrid e-learning system. In addition, the perception that medical apps create a social gap between lecturers and learners was decreased in phase 2, which could be attributed to the active dependence on e-learning and medical apps after the lockdown [13].

On the other hand, a minority of participants in phases 1 and 2 indicated that medical apps foster independence in their studies, accounting for 40% and 48.7%, respectively. This finding aligns with Saudi students’ attitudes towards medical apps, with the majority of participants expressing that while medical apps are convenient to download and use, they cannot rely solely on them to cover their medical curriculum [11].

### Limitations of medical app usage

Lack of knowledge about which app to download was the main obstacle hindering students from using medical apps, particularly among females. Limited Internet access and the high cost of apps were also common limitations cited by the participants. Previous research on the challenges encountered by students agreed with our results that the most commonly reported obstacle was a lack of knowledge about which app to use [15]. Providing advice to medical students from experienced seniors and faculty members regarding the latest advancements and updates in medical applications may mitigate this challenge [15].

As for students who had never paid for an app, they reported that their devices could not support the installation of new applications. This could be one reason that prevented them from paying money to subscribe. However, the biggest reason that should not be overlooked is that the subscription fees for valuable and reliable apps are very high and unaffordable for students, especially in light of the poor economic status of low- and middle-income countries, which makes buying mobile apps a dispensable luxury [15].

### Limitations of the study

Although our study provides valuable insights, it has several limitations. The low participation of male students in the first phase of data collection was a key limitation, with female students accounting for 71.3% of the responses. We faced challenges in reaching male students during the data collection. Additionally, our study lacked a multicenter design, and the sample was drawn from a single medical school, which may limit the generalizability of our findings to all Egyptian medical students. Nevertheless, our study is the first of its kind in Egypt and provides a valuable foundation for future research on a larger scale.

## Conclusion

The use of smartphones and mobile medical apps increased significantly after the COVID-19 lockdown. In both phases of the study, most students showed a positive attitude towards medical apps regarding confidence, flexibility, and completing assignments. Although the majority agreed that it was a flexible way of learning, they also agreed that these apps could not be solely relied on during medical study. Not knowing which app to use, limited Internet access, and high costs were the main obstacles that prevented students from using the apps more widely.

## Data Availability

The datasets used and analyzed during the current study are available from the corresponding author upon reasonable request.

## Abbreviations

Apps: Applications
